# Rebuttal to Douglas and Elliott

**DOI:** 10.1007/s10838-022-09616-3

**Published:** 2022-05-11

**Authors:** Robert Hudson

**Affiliations:** Department of Philosophy, University of Saskatchewan, 9 Campus Drive, S7N 5A5 Saskatoon, Saskatchewan Canada

## Abstract

In “Should We Strive to Make Science Bias‑Free? A Philosophical Assessment of the Reproducibility Crisis”, I argue that the problem of bias in science, a key factor in the current reproducibility crisis, is worsened if we follow Heather Douglas and Kevin C. Elliott’s advice and introduce non-epistemic values into the evidential assessment of scientific hypotheses. In their response to my paper, Douglas and Elliott complain that I misrepresent their views and fall victim to various confusions. In this rebuttal I argue, by means of an examination of their published views, that my initial interpretation of their work is accurate and that, in their hands, science is generally prone to deviations from truth.

Upon reading Heather Douglas and Kevin C. Elliott’s response to my paper, I was pleased to see that we largely agree on the definition of ‘bias’. On their view, bias is “a systematic deviation from truth (or from accuracy)”. By comparison, I say: “a process of (statistical) reasoning is biased if it is influenced by factors … that make the conclusion of this reasoning less likely to be true” (Hudson 2021, 399). I call such factors ‘non-epistemic’, a suggestion echoed by Steel (2017) according to whom “epistemic values … are typically thought to be distinguished by their tendency to promote the acceptance or belief of true claims” whereas “non-epistemic values are … types of values that are not truth promoting” (50). Elliott, too, distinguishes epistemic from non-epistemic values, with the former concerning “accurate predictions and logical consistency” and the latter dealing with “ethical or political or religious values” (2017, 12). As such, if one allows non-epistemic values to influence reasoning (and henceforth following Elliott I use the term ‘value’ as short for ‘non-epistemic value’), it follows that this reasoning will tend to veer from the truth, from predictive accuracy, or even from logical consistency. That is, such reasoning will be prone to bias because it is value-laden.

Douglas and Elliott claim that that this inference misrepresents their views. They comment, “we reject Hudson’s inference that if we accept roles for non-epistemic values in scientific reasoning, we must embrace bias in science”, and assert that one of my “most important confusions is [my] conflation of bias and value-ladenness”. To be clear: someone’s reasoning can be biased, can be value-laden, but lead to a true conclusion. This is because bias involves a tendency to deviate from truth or make the conclusion of reasoning less likely to be true, not the stronger effect of leading consistently to falsity. So, take the cases Douglas and Elliott cite of value-ladenness without bias, cases of anti-sexism that have improved the accuracy of claims made in specific historical cases of primatology, cardiology, social science and archeology. Although, in these cases, anti-sexism possesses a clear epistemic benefit in terms of revealing problematic research assumptions apparently based on sexist attitudes, it would be a stretch to say that anti-sexism always has such clear epistemic benefits. This is simply because anti-sexism, as a moral standard, is largely irrelevant to epistemic matters. In terms of developing research methodologies that generate empirically verified, theoretical hypotheses, science students are better off learning mathematics, statistics and various experimental or laboratory skills rather than studying the moral underpinning of anti-sexism. In other words, if science students neglect their study of the core methods of science in deference to studying anti-sexism, they are much less likely discover true theories about the natural world. In other words, their research would be biased according to the standards set by Elliott, Douglas, Steel and myself.

A more accurate description of what is going on in the above cases of sexist science is to say that anti-sexism in those cases is, to borrow a phrase from (Steel 2010), an ‘extrinsic’ epistemic value in that, in the cases at hand, anti-sexist attitudes “promote the attainment of truth without themselves being indicators or requirements of truth” (18). Or, alternatively, as per Rooney (2017), anti-sexism is working in these cases as a ‘meta-epistemic value’ “to the extent that feminist critiques of science are helping to uncover limiting effects of traditional cultural values” (41–42). From this perspective, Douglas and Elliott are correct when they say that, “even when values influence scientific inference, [this] does not necessarily cause bias”, if they mean that, in certain cases, values promote the attainment of truth. But, of course, values do not promote the attainment of truth, generally speaking — they are not designed to do that since they are normative prescriptions on a vast number of issues other than truth-seeking. Accordingly, as regards their first criticism of my paper, value-ladenness does involve bias, in general, though it may nevertheless be truth-conducive in particular cases.

Douglas and Elliott’s second criticism of my paper is that, in portraying values as serving an evidential role in situations of inductive risk, I am describing their views in a “deeply misleading way”. Specifically, contrary to what I suggest, Douglas “does *not* think values serve as evidence; instead, they establish the *amount* of evidence needed in order to accept a scientific conclusion” (their italics). Again, Douglas does not see “values as ‘evidential’ or that they could fill an evidential gap” and she “has carefully avoided describing values as playing this role in scientific inference”. To say that values could serve as evidence is to ascribe to values a *direct* role in science: in such a role, values act as “stand-alone reasons to motivate [scientific] choices”, providing “warrant or reason”, in themselves, for such choices (Douglas 2009, 96). By comparison, values play an *indirect* role in science if they do not act as such stand-alone reasons. Rather, in cases where the extant evidence is “incomplete … and thus there is uncertainty” about what choice to make, “values serve a crucial role of helping us determine whether the available evidence is sufficient for the choice”, taking into consideration “the potential consequences of a wrong choice” (96–97). Here, “the adequacy of evidential support involves (or should involve) social and ethical values” (Douglas 2017, 82).

Now, it is worth pointing out that Douglas’ direct/indirect distinction has undergone sustained criticism in the values and science literature. In suggesting that an indirect role for values in science does not compromise epistemic standards, whereas a direct role for values does, Douglas intends to ensure the epistemic integrity of science by restricting values to an indirect role. But in a hypothetical case proposed by Steel and Whyte (2012, 171), cited by both Elliott (2013, 378) and Resnik and Elliott (2019, 4), an indirect role for values can have problematic epistemic consequences. In that case, given the uncertainty of experimental results relating to the adverse effects of a favoured pharmaceutical product, a company can raise the evidential standard for demonstrating the presence of such effects, effectively burying any evidence about the adverse nature of the product, which Steel and Whyte describe as a violation of severity (i.e., experiments will not reveal adverse reactions even if they are present, since the standards for showing this are too high). The problematic influence of values on scientific reasoning, given Douglas’ direct/indirect distinction, extends even deeper once one considers the methodological decisions made early in a research program, where on Douglas’ view values can legitimately play a direct role (see Elliott 2013, 376, citing Douglas 2008 and 2009). As Resnik and Elliott (2019, 4–5) point out, allowing values to influence these early decisions can lead to ‘skewed’ or ‘biased’ research. A related observation is made by Bluhm (2017) who notes that if ethical values are allowed to influence prior methodological choices, this will in turn “shape the data collected” and thus “the evidence available to confirm … hypotheses” (208–209). Put another way, values will play a role in how empirical data are ‘characterized’, in how the evidential impact of data is interpreted (see Elliott 2011a, 65–69, 2011b, 306, and Elliott and Richards 2017, 3–4). Douglas recognizes the problems inherent in letting values play a direct role in the characterization of data – it “would allow scientists to reject data if they did not like it” (2009, 102) – but admits that similar characterizations occur even where values play an indirect role (106). To be sure, Douglas describes the epistemic effects of values in indirect roles as occurring in ‘borderline cases’, such as with the carcinogenic nature of dioxin. However, science is rife with borderline cases, and so it follows that “if nonepistemic values help to determine standards of evidence”, as they do when playing an indirect role, “they can end up permeating the entire research program” (Elliott 2011a, 69). In Douglas’ words, “science is saturated with values” (Douglas 2009, 113) to such an extent that “social and ethical values are a well-justified part of scientific reasoning” (Douglas 2017, 92).

Consequently, I do not think my concern about the epistemic hazard of introducing values in scientific reasoning, even where the role of such values is indirect, is off-the-mark. In fact, it is explicitly shared by Resnik and Elliott who assert, concerning the sorts of problems expressed above, that “the distinction between direct and indirect roles for values is relatively unhelpful for preventing problems of this sort” (2019, 5). They continue: “clearly, allowing non-epistemic values to supplant empirical evidence threatens the integrity, objectivity, and reliability of research” (6).

This takes us to Douglas and Elliott’s third criticism of my paper, that I have presented “a false dichotomy, with policy makers making pragmatic decisions to accept hypotheses because of values as one option and scientists making evidential decisions to believe hypotheses because of values as the other option”. The third option, which they endorse, is to view scientists as “[drawing] conclusions on the basis of ethical and social values” where scientists “treat these decisions as pragmatic rather than as evidential”, all the while being clear about “the value judgments used to come to their conclusions”. For this third option to be feasible, Douglas and Elliott claim that a distinction needs to be drawn between “[accepting] a hypothesis as a basis for policy making” versus “considering whether or not to believe that the hypothesis is true”, where “ethical or social values are relevant” to the former in contrast to the latter where “ethical values are logically irrelevant and have no legitimate role to play because belief is directed only toward achieving truth” (quoting Elliott and Willmes 2013, 812).

It turns out that the distinction between belief and acceptance drawn in Elliott and Willmes (2013) is useful in explaining how bias results from value-laden science. In that paper, one of their “lessons” is that, when dealing with a science in a state of prevailing underdetermination, “acceptance is a much more appropriate cognitive attitude to adopt than belief” (812). This corresponds to Douglas’ suggestion that, where there is uncertainty in the evidential support for an hypothesis, values can play an indirect role in guiding us to accept, though not believe, this hypothesis. To illustrate their view, Elliott and Willmes consider the case of C. Owen Lovejoy’s male provisioning hypothesis in paleoanthropology which, they argue, is in just such a state of underdetermination (they say, “it is arguably inappropriate for scientists to adopt the attitude that the male provisioning hypothesis is true or even highly probable”, 814). In a state of underdetermination, and given that paleoanthropologists enjoy “scientific authority” that allows them to “influence contemporary conceptions of human beings and their social organization” (813), Elliott and Willmes suggest, “at a minimum, … if the male provisioning hypothesis is likely to contribute to social attitudes that cause harm to women, that may provide reasons, though perhaps not decisive reasons, not to investigate it as aggressively as one otherwise would” (815). Clearly, if one declines to take some hypothesis seriously because of its potentially untoward social or moral implications, then one may as well have found evidence against it: values are playing the role normally played by evidence, and the distinction between acceptance and belief makes no difference. Moreover, given that a state of uncertainty, a state of underdetermination, is the norm for any scientific hypothesis of non-trivial scope, the result is that science, writ large, and not just policy-relevant science, is subject to the yoke of social and moral sensitivities, with values filling the spaces left vacant by the absence of empirical facts.

Finally, we have the matter of the reproducibility crisis, and what Douglas and Elliott see as reasonable responses to this crisis. It is heartening to see their approval of registered reporting, though in my paper I never denied that this is an option for them. In fact there are a lot of options for them in responding to the crisis. Where an experimental result fails to be replicated, this means there is uncertainty about whether one should endorse the result, and so for Douglas and Elliott the opportunity arises to implement one’s favored (ethical or social) values in determining whether to accept the result, perhaps by lowering one’s standards of evidence.

However, Douglas and Elliott find my suggestion that it is problematic to liberally modify evidential standards based on valuational considerations to be a “strange critique of their views”. This is because they do not see any feasible alternative to allowing such flexibility in standards, such as insisting on high standards (which could never be met) or standards that are universally correct (which could never be found). Nevertheless, in alignment with the views of John (2015), they see a threat to the transparency of scientific decision-making if evidential standards are allowed to vary depending on the context — if, as John says, scientists “say one thing in Brussels, another thing in a journal, and a third thing in a newspaper editorial” (91) —, leading to potential confusion in the public. In response, Douglas and Elliott entertain the option to “conventionally [maintain] fixed standards of evidence” as a way to address what John describes as “a complex coordination problem” (94), the problem of how to ensure the “efficient co-ordination of experts’ claims and non-experts’ practical needs” (93). Ironically, the value-free ideal becomes, for John — and here Elliott apparently concurs —, a way to solve this coordination problem, albeit now defended as a social value (see Douglas 2009, 135–136, for a similar sentiment).

It is not surprising, then, that Douglas and Elliott ultimately abandon the suggestion to “maintain fixed standards of evidence” in addressing the problem of bias, in deference to the strategy of registered reporting. And, in general, they view the value-free ideal as utterly unhelpful in addressing the problem of bias in science where this bias is “traceable to values in science”. Here, I completely agree with them. Once values “‘infect’ the science that makes policy” (Douglas 2009, 135), the special tools scientists use to make “accurate predictions” and ensure “logical consistency” have little use. In fact, we need not even be talking about science anymore as the pressing issues of “how best to define and manage conflicts of interest, how to mitigate career pressures to publish, or how to decide which research to fund”, and so on, afflict any academic endeavor, scientific and otherwise. In other words, if the “way forward” in “protecting and promoting scientific integrity” is to turn to “the literature on research ethics” (Resnik and Elliott 2019, 6), this exempts us from the need to investigate a prevailing state of underdetermination in science by learning more mathematics or statistics, improving our experimental or laboratory skills, or using any of the other ‘value-free’ elements of scientific methodology.

## References

[CR1] Bluhm, R. 2017. Inductive Risk and the Role of Values in Clinical Trials. In K. Elliott and T. Richards (Eds.), *Exploring Inductive Risk: Case Studies of Values in Science* (pp. 193–212). New York: Oxford University Press.

[CR4] Douglas, H. 2008. The role of values in expert reasoning. *Public Affairs Quarterly*, 22, 1–18.

[CR6] Douglas H (2009). Science, Policy, and the Value-Free Ideal.

[CR8] Douglas, H. 2017. Why Inductive Risk Requires Values in Science. In K. Elliott and D. Steel (Eds.), *Current Controversies in Values and Science* (pp. 81–93). New York: Routledge.

[CR10] Elliott, K. 2011a. *Is a Little Pollution Good for You? Incorporating Societal Values in Environmental Research*. New York: Oxford University Press.

[CR12] Elliott, K. 2011b. Direct and Indirect Roles for Values in Science. *Philosophy of Science*, 78, 303–324.

[CR14] Elliott, K. 2013. Douglas on values: From indirect roles to multiple goals. *Studies in History and Philosophy of Science Part A*, 44, 375–383.

[CR16] Elliott, K. 2017. *A Tapestry of Values: An Introduction to Values in Science*. New York: Oxford University Press.

[CR18] Elliott, K., and D. Willmes. 2013. Cognitive Attitudes and Values in Science. *Philosophy of Science*, 80, 807–817. 10.1016/j.shpsa.2015.05.011 26386530

[CR20] Elliott, K., and T. Richards. 2017. Exploring Inductive Risk: An Introduction. In K. Elliott and T. Richards (Eds.), *Exploring Inductive Risk: Case Studies of Values in Science* (pp. 1–13). New York: Oxford University Press.

[CR23] Hudson, R. 2021. Should We Strive to Make Science Bias–Free? A Philosophical Assessment of the Reproducibility Crisis. *Journal for General Philosophy of Science, *52, 389–405. 10.1007/s10838-020-09548-w PMC8550477 34720421

[CR25] John S (2015). Inductive Risk and the Contexts of Communication. Synthese.

[CR26] Resnik, D., and K. Elliott. 2019. Value-entanglement and the integrity of scientific research. *Studies in History and Philosophy of Science Part A*, 75, 1–11. 10.1016/j.shpsa.2018.12.011 PMC12614358 31426942

[CR28] Rooney, P. 2017. The Borderlands Between Epistemic and Non-Epistemic Values. In K. Elliott. and D. Steel, eds., *Current Controversies in Values and Science* (pp. 31–45). New York: Routledge.

[CR31] Steel, D. 2010. Epistemic Values and the Argument from Inductive Risk. *Philosophy of Science*, 77, 14–34.

[CR33] Steel, D. 2017. Qualified Epistemic Priority Comparing Two Approaches to Values in Science. In K. Elliott, and D. Steel (Eds.), *Current Controversies in Values and Science* (pp. 49–63). New York: Routledge.

[CR36] Steel D. and K. Whyte. 2012. Environmental Justice, Values, and Scientific Expertise. *Kennedy Institute of Ethics Journal*, 22, 163–182. 10.1353/ken.2012.0010 23002582

